# Editorial: Environment, Art, and Museums: The Aesthetic Experience in Different Contexts

**DOI:** 10.3389/fpsyg.2021.675165

**Published:** 2021-04-29

**Authors:** Stefano Mastandrea, Pablo P. L. Tinio, Jeffrey K. Smith

**Affiliations:** ^1^Department of Education and Laboratory of Experimental Psychology, Roma Tre University, Rome, Italy; ^2^Department of Educational Foundations, Montclair State University, Montclair, NJ, United States; ^3^College of Education, University of Otago, Dunedin, New Zealand

**Keywords:** aesthetic experience, environment, museum, art appreciation, landscapes

The aesthetic experience may be defined as people's interactions with, and reactions to, objects, places, but also to the environment. Most psychological perspectives on the aesthetic experience argue that it results from the coordination of different mental processes such as perception, attention, memory, imagination, thought, and emotion. Physiological and neurological responses are also involved. Aesthetic experiences can take place while we observe works of art in museums and galleries as well as in other contexts such as natural and built environments. Looking at a landscape, walking in a park, meeting people in a square, and walking into a building that is architecturally appealing are examples of natural and built environments where we can experience beauty, pleasure, attraction, and interest, among other aesthetic reactions.

Research on aesthetic experiences has a long history, and in recent decades, the field has experienced tremendous growth in the number of empirical studies conducted. One of the areas that researchers have yet to fully address is the influence of the context (natural and built environments) on aesthetic experiences. We refer to context according to three broad categories: *Context as natural environments, context as built environments*, and *environments for aesthetic experiences*.

## Context as Natural Environments

People show a basic tendency to associate the natural environment with positive evaluations. According to an evolutionary explanation known as the *biophilia hypothesis* (Kellert and Wilson, 1993), human beings, who have evolved in natural environments, have developed an innate tendency to positively respond to nature as a consequence of an adaptation process.

## Context as Built Environments

Urban environments, architecture, and buildings that have been systematically designed for both function and aesthetics can affect people's behaviors and social relationships (Mastandrea et al., 2009).

## Environments for Aesthetic Experiences

Museums can be considered as built environments, and some museums have even been designed so that they themselves could be seen as works of art, as aesthetic objects to be appreciated. These include specific elements of museums, from the halls to the artworks, from the arrangement of art in an exhibition, to the paths that visitors follow and the way that objects are displayed. These design elements can also influence visitors' enjoyment of the art collection (Tinio and Smith, 2014; Mastandrea et al., 2019).

We have received interesting contributions from scholars with different backgrounds, leading to a rich tapestry of offerings. We can synthesize the different topics into three broad categories: *Aesthetic experience in museums and art exhibitions, Art appreciation in ecological settings and different art contexts*, and *Environment and landscapes*.

## Aesthetic Experience in Museums and Art Exhibitions

Regarding this topic, Myszkowski and Zenasni, in “Using Visual Aesthetic Sensitivity Measures in Museum Studies,” provide a history and an overview of visual aesthetic sensitivity as well as how it is measured and what it can tell us about individual differences in experiences and judgements of art. Importantly, the authors make a convincing argument of why visual aesthetic sensitivity measures should be implemented in research in museums.

Krukar and Dalton in “How the Visitors' Cognitive Engagement is Driven (but not Dictated) by the Visibility and Co-Visibility of Art Exhibits,” asked participants to wear mobile eye-tracking while visiting an art exhibition with different spatial locations of the artworks. The exhibition's visual properties influenced the experience of museum visitors. More visible locations attracted more attention and the amount of attention improved the recognition and memory of pictures.

Annechini et al., in “Aesthetic attributes of museum environmental experience: a pilot study with children as visitors,” highlighted the importance of the restorative aspect of a museum environment for children. They appraised the impact of museum environment on children during museum learning and experiential activities. In a case study, authors tried to understand and evaluate the museum impact on learning and experiential activities in children in the museum of contemporary art, MART, in Rovereto, Italy. Findings show that for most children, the MART museum (and for extension museums in general) provides a sense of relaxation and well-being during the museum visit and the aesthetic experience.

Bertamini and Blakemore, in “Seeing a work of art indirectly: When a reproduction is better than an indirect view, and a mirror better than a live monitor,” used a survey and a set of hypothetical questions to explore three different alternatives of museum or exhibition: seeing an optical reflection (using a mirror), seeing a video screening (a closed-circuit camera) or seeing a reproduction. There was an overall preference for seeing a reproduction as opposed to an optical or digital image. Contrary to the idea that the original is always superior to a copy, many people felt that a direct view of a copy is a preferable experience than an indirect view.

Pelowski et al. in “Does Gallery Lighting Really have an Impact on Appreciation of Art? An ecologically-valid study of lighting changes and the assessment and emotional experience with representational and abstract paintings,” presented a selection of realistic and abstract original artworks under three different lighting intensity/temperature conditions. Findings show that for both realistic and abstract paintings, the light changes in the gallery settings did not show significant effects on the evaluation and emotional experience within the artworks.

## Art Appreciation in Ecological Settings and Different Art Contexts

Regarding the second topic we have three interesting articles. In “Communication and Meaning-Making are Central to Understanding Aesthetic Response in Any Context,” Dolese and Kozbelt advocate for the use of a framework developed by Grice in helping us understand how to communicate via art, whether that communication is from artist to viewer, curator to visitor, or viewer to oneself. They discuss issues of what art means to individuals, and how they go about determining what that meaning is.

Estrada-Gonzalez et al. take a fascinating look at how we look at original artworks vs. computer reproductions of art in “Viewing Art in Different Contexts.” They employ eye movement cameras to record fixations of works of art in a museum setting vs. computer reproductions that either used the same size image for all works, or a roughly proportional representation of the works. Their findings are complex, but generally indicate that the physical characteristics of the painting along with whether the image was in a gallery or on computer made a difference in viewing.

Carbon in “Ecological Art Experience: How we can gain experimental control while preserving ecologically valid settings and context,” compared art experience in different art settings while participants observed paintings by Pollock and Rothko at different viewing distances. Liking of painting was correlated with farther distances, but insights of the artworks were not correlated to liking. Moreover, among the evaluative variables used by participants, interestingness, and powerfulness, were considered as predictors of how much people like paintings.

## Landscapes and Environment

In this third topic, Law et al. in “Viewing natural landscapes is more stimulating than scrambled images after a stressor: a cross-disciplinary approach,” show that viewing landscape paintings increased psycho-physiological responses (cortisol level, pupil size), compared to viewing scrambled images obtained from the correspondent landscape artworks. While viewing landscapes the average pupil size was bigger compared to scrambled pictures; it is known that increased pupil size is related to augmented cognitive engagement, attention, and arousal.

Løvoll et al. in “Feeling at Home in the Wilderness: Environmental Conditions, Well-Being and Aesthetic Experience,” conducted an original experience. Participants (47) undertook a 5-day, winter, wilderness adventure training with the aim to challenge wilderness and leadership skills under two different extreme weather conditions. Findings show that there was a correlation between the evaluation of the sentence “I felt at home in nature” and satisfaction with life and personal growth trait measures, mainly during sunny and cold weather conditions, and on the contrary not significant in stormy and wet weather in a mountain forest. The finding related to feelings and well-being are explained in term of relationship to self-awareness.

The studies presented took into consideration several different contexts: laboratory, museum, natural environment. These different approaches and settings can allow us to get more insight on the aesthetic experience while observing original arts, digital reproductions, nature and landscapes.

## Author Contributions

SM conceived the idea of this Research Topic. All authors listed contributed to the work and approved the submitted version.

## Conflict of Interest

The authors declare that the research was conducted in the absence of any commercial or financial relationships that could be construed as a potential conflict of interest.
